# Computational estimation of quality and clinical relevance of cancer cell lines

**DOI:** 10.15252/msb.202211017

**Published:** 2022-07-13

**Authors:** Lucia Trastulla, Javad Noorbakhsh, Francisca Vazquez, James McFarland, Francesco Iorio

**Affiliations:** ^1^ Human Technopole Milano Italy; ^2^ Open Targets Cambridge UK; ^3^ Broad Institute of MIT and Harvard Cambridge MA USA; ^4^ Department of Medical Oncology Dana‐Farber Cancer Institute Boston MA USA; ^5^ Present address: Kojin Therapeutics Boston MA USA

**Keywords:** cancer cell lines, computational biology, drug discovery, personalised medicine, pharmacogenomics, Cancer, Computational Biology, Pharmacology & Drug Discovery

## Abstract

Immortal cancer cell lines (CCLs) are the most widely used system for investigating cancer biology and for the preclinical development of oncology therapies. Pharmacogenomic and genome‐wide editing screenings have facilitated the discovery of clinically relevant gene–drug interactions and novel therapeutic targets via large panels of extensively characterised CCLs. However, tailoring pharmacological strategies in a precision medicine context requires bridging the existing gaps between tumours and *in vitro* models. Indeed, intrinsic limitations of CCLs such as misidentification, the absence of tumour microenvironment and genetic drift have highlighted the need to identify the most faithful CCLs for each primary tumour while addressing their heterogeneity, with the development of new models where necessary. Here, we discuss the most significant limitations of CCLs in representing patient features, and we review computational methods aiming at systematically evaluating the suitability of CCLs as tumour proxies and identifying the best patient representative *in vitro* models. Additionally, we provide an overview of the applications of these methods to more complex models and discuss future machine‐learning‐based directions that could resolve some of the arising discrepancies.

## Graphical abstract

Cancer cell lines (CCLs) are widely used for analysing cancer and identifying therapies. This Review discusses the limitations of CCLs in representing tumours and considers computational methods that can systematically evaluate the suitability of CCLs as tumour proxies.

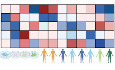

## Cancer cell lines: a mainstay for cancer biology, drug discovery and large‐scale multi‐omic data generation

**Figure 1:**
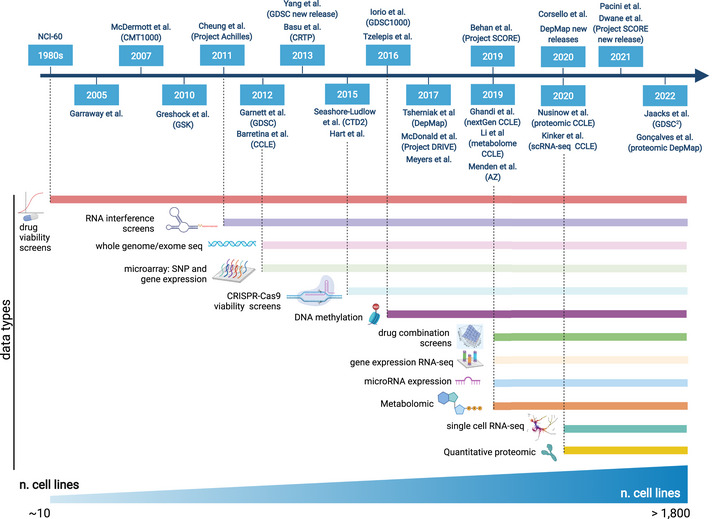
Major public cell line‐based data sets with corresponding omics and reference publications The horizontal bars indicate the data type/omic type availability. Created with BioRender.com.

Since the first cultured cell line was established in 1951 from Henrietta Lacks' cervical cancer cells (Scherer *et al*, 1953), the use of immortalised cell lines as cancer *in vitro* models has become a pivotal tool for studying primary tumours. Cancer cell lines (CCLs) are widely used for therapy discovery, as they are easily amenable to experimental manipulation, and suitable for high‐throughput screens, supporting the generation of large‐scale perturbation data sets (McDonald *et al*, 2017; Meyers *et al*, 2017; Tsherniak *et al*, 2017; Behan *et al*, 2019), as well as comprehensive multi‐omic characterizations (Gillet *et al*, 2013; Ghandi *et al*, 2019; Fig 1).

Currently, the use of large‐scale cell‐line‐based multi‐omic data sets is having a major impact on drug discovery and repositioning, facilitating the identification of genetic linkages between candidate drug targets and disease biology, thus increasing the likelihood of investigative compounds to progress through the different phases of clinical development (Wilding & Bodmer, 2014; Nelson *et al*, 2015; Corsello *et al*, 2020; Francies *et al*, 2020). Starting from the pioneer NCI‐60 panel, created in the 1980s (Shoemaker, 2006) and aimed at identifying compounds with tumour‐type‐specific growth‐inhibitory effects across 60 CCLs, next‐generation high‐throughput techniques have given rise to large‐scale pharmacogenomic screens (Sharma *et al*, 2010), in the attempt to dissect the relationship between cell viability reduction upon compounds' treatment and genetic features.

Besides extensively characterisations of the NCI‐60 panel (Garraway *et al*, 2005), tremendous effort has been and is still being made to assemble increasingly large CCL drug response data sets. These embody quite comprehensive tumour molecular heterogeneity together with viability reduction measurements of thousands of *in vitro* models in response to treatment with hundreds of compounds. Examples include the *Cancer Cell Line Encyclopedia* (CCLE; Barretina *et al*, 2012), the *Genomics of Drug Sensitivity in Cancer* (GDSC; Garnett *et al*, 2012; Yang *et al*, 2013), the *Centre for Molecular Therapeutics 1000* (CMT1000; McDermott *et al*, 2007), *Cancer Target Discovery and Development* (CTD (Basu *et al*, 2013) and CTD^2^ (Seashore‐Ludlow *et al*, 2015)) and a study from (Greshock *et al*, 2010). In addition, comprehensive drug response data sets have been recently expanded to incorporate non‐oncology drugs (Corsello *et al*, 2020), and combinatorial treatments (Menden *et al*, 2019; Jaaks *et al*, 2022).

In parallel, CCL characterizations have expanded in the direction of multi‐omic data assembly to reveal regulatory mechanisms associated with cancer vulnerabilities arising from cancer driver genomic (as well as, epigenetic and transcriptomic) alterations. For instance, CCLE moved beyond the initial genomic and transcriptomic space and characterised RNA splicing, DNA methylation, microRNA expression, global histone modifications, proteomic and metabolomic quantitative profiles in more than 1,000 CCLs from multiple lineages and ethnicities (Ghandi *et al*, 2019; Li *et al*, 2019; Nusinow *et al*, 2020; preprint: Goncalves *et al*, 2022). Among those, a subset of 198 CCLs in 22 cancer types have been recently profiled by single‐cell RNA‐seq to study intra‐tumour heterogeneity (Kinker *et al*, 2020).

Various computational approaches have been used to jointly analyse these drug response data sets and the comprehensive multi‐omic characterisations of the CCLs, revealing molecular features that are informative and predictive of drug response, most often based on stratifying CCLs on the presence/absence of individual molecular features. This has allowed recovering established and identifying novel genomic (Basu *et al*, 2013; Seashore‐Ludlow *et al*, 2015; Iorio *et al*, 2016; Jaaks *et al*, 2022) as well as transcriptional (Garcia‐Alonso *et al*, 2018; Jaaks *et al*, 2022) markers of drug sensitivity, leading to new testable hypotheses and clinical trials. For instance, Ewing's Sarcoma lines were found to be hypersensitive to PARP inhibitors (Gill *et al*, 2015), leading to the proposed use of these inhibitors in combination with chemo/radiotherapy (Vormoor & Curtin, 2014). Canakinumab and spartalizumab are undergoing phase 1 clinical trial as a combinatorial treatment for clear cell renal carcinoma patients (NCT04028245) and entinostat (histone deacetylase inhibitor) is undergoing phase 2 in the treatment of neuroendocrine tumours (NCT03211988). Finally, CHEK1 inhibitors were found to act synergistically with chemotherapy (irinotecan) in microsatellite‐stable and KRAS‐TP53 double‐mutant colon cancer cells, both *in vitro* and *in vivo* (Jaaks *et al*, 2022).

Simultaneously, large‐scale RNA interference (RNAi) (Cheung *et al*, 2011; McDonald *et al*, 2017; Tsherniak *et al*, 2017) and genome‐wide CRISPR‐Cas9 knockout screens (Hart *et al*, 2015; Tzelepis *et al*, 2016; Meyers *et al*, 2017) performed on large panels of CCLs enabled the systematic identification of cancer genetic dependencies (i.e. genes necessary for cancer cell proliferation and survival, also called fitness genes) (Grimm, 2004). With higher efficiency and precision compared with RNAi (Evers *et al*, 2016), CRISPR‐based studies have elucidated the landscape of cancer vulnerabilities and unveiled novel and therapeutically exploitable synthetic‐lethalities (Chan *et al*, 2019), allowing the development of advanced bioinformatics methods for the identification and the prioritisation of new candidate therapeutic targets on a genome scale (Behan *et al*, 2019).

Increasing the level of complexity, more recent *in vitro* screens are focussing on digenic dependencies, uncovering compensatory relationships between pairs of genes and are starting to identify interactions that are synthetic lethal for cancer cell survival, and most often involve specific paralogous genes (Ito *et al*, 2021; Thompson *et al*, 2021). In addition, CCL‐based post‐perturbational transcriptomic data sets such as the Connectivity Map (Lamb, 2007; Bush *et al*, 2017; Ye *et al*, 2018) and related Web resources (Stathias *et al*, 2020) have been pivotal for computational drug discovery and repositioning (Pushpakom *et al*, 2018), and are now being increasingly assembled also at single‐cell resolution (McFarland *et al*, 2020; Srivatsan *et al*, 2020).

This ecosystem of CCL data sets is publicly accessible, actively curated and allows generating new hypotheses about the biology of cancer, its dependencies and response to therapy (Table 1). For instance, Cellosaurus (Bairoch, 2018) provides curated CCL metadata resources across multiple species. COSMIC (Tate *et al*, 2019) includes the Cell Lines Project dataset (Iorio *et al*, 2016), which collects exome sequencing data and molecular profiling of more than 1,000 CCLs. cBioPortal (Gao *et al*, 2013) allows users to interactively explore multidimensional cancer genomic and clinical data sets, including data visualisation and analytical options across genes, samples and data types, gathering both CCL and patient tumour information. The GDSC (Yang *et al*, 2013) and GDSC^2^ (Jaaks *et al*, 2022) databases are large public resources of drug sensitivity data derived from treating more than a thousand CCLs with hundreds of individual and pairs of compounds, respectively. These resources are also paired with Web portals equipped with interactive data exploration tools, aiming at facilitating the discovery of statistical associations between molecular features and differential treatment response to single or combinatorial therapies. The Cancer Dependency Map has continued to generate and refine data from the characterisation of increasingly larger CCL collections, now accounting for more than 1,800 *in vitro* models, and making the corresponding omics and CRISPR‐screening data available pre‐publication. Similarly, the Cell Model Passports portal (van der Meer *et al*, 2018) includes highly curated multi‐omic and clinical data sets derived from the characterisation of more than 1,900 CCLs and organoids. The Project Score (Dwane *et al*, 2021) database allows the exploration of systematic genome‐scale CRISPR‐Cas9 dropout screen results in a variety of CCLs. Finally, the Online Gene Essentiality Database (Gurumayum *et al*, 2021) contains gene fitness data for 91 species, encompassing more than 500 CCLs.

**Table 1:** Portals providing access to large CCL‐based data sets and related *in vitro* models' curated annotations.

Portal name	URL	Available info
Cellosaurus	https://web.expasy.org/cellosaurus/	CCL names with synonyms, sex and age of the donor, and molecular charachteristics (MSI, doubling time etc).
		Engineering procedure (gene KO or insertion), resistance to drug, known contaminations.
COSMIC	https://cancer.sanger.ac.uk/cosmic	Catalogue of cancer somatic mutations: variant type, gene fusions, CN variants, drug resistant mutations, GE and HypMet effects.
	https://cancer.sanger.ac.uk/cell_lines	CCLs' exome sequencing and other molecular profiles.
cBioPortal	https://www.cbioportal.org/	Interactive exploration of genetic, epigenetic, gene expression, proteomic events and clinical data. Connection to disrupted pathways.
GDSC	https://www.cancerrxgene.org/	CCLs' drug sensitivity and molecular markers of drug response.
GDSC2	https://gdsc‐combinations.depmap.sanger.ac.uk/	CCLs' drug combination sensitivity and related molecular markers.
DepMap	https://depmap.org/portal/	Portal collecting multi‐omic data from the characterisation of 100s of CCLs (maintained at the Broad and other institutes).
		CCLs' molecular, drug sensitivity, gene essentiality (from CRISPR‐Cas9 and RNAi screens) profiles.
CellModelPassport	https://cellmodelpassports.sanger.ac.uk/	Portal with multi‐omic data from the characterisation of 100s of CCLs (maintained at the Wellcome Sanger institute).
		CCLs' molecular, drug sensitivity, gene essentiality (from CRISPR‐Cas9 screens) profiles.
ProjectScore	https://score.depmap.sanger.ac.uk/	Systematic genome‐scale CRISPR‐Cas9 drop‐out screens with exploration tools.
Online Gene Essentiality Database	https://v3.ogee.info/	CCLs' gene essentiality profiles (from CRISPR‐Cas9 and RNAi screens).

Despite initial concerns about inter‐study reproducibility (Haibe‐Kains *et al*, 2013), this plethora of resources has been proven consistent across institutes and publications, from a pharmacogenomic point of view (Cancer Cell Line Encyclopedia Consortium & Genomics of Drug Sensitivity in Cancer Consortium, 2015; Geeleher *et al*, 2016; Haverty *et al*, 2016), as well as when considering drug response profiling (Mpindi *et al*, 2016) and CRISPR‐Cas9 screens (Dempster *et al*, 2019). This agreement across studies has allowed their integration (Pacini *et al*, 2021), paving the way to large unified resources and inter‐study/institute Cancer Dependency Maps (Boehm *et al*, 2021). Compared with more recent cancer models such as patient‐derived xenografts (PDx) and patient‐derived organoids (PDO), the scalability and cost efficiency of CCLs is reflected by the larger volume and diversity of available data (Feng *et al*, 2021). Hence, it is likely that for the foreseeable future, CCLs will remain the main source of information for genomics‐guided and data‐driven preclinical development of cancer therapies (Francies *et al*, 2020), and for the discovery and validation of cancer genetics dependencies (Lin & Sheltzer, 2020). Nonetheless, CCLs have intrinsic and unsurmountable limitations, including the fact that they are cultured in 2D flat dishes, growing in cell culture media and lacking matching tumour microenvironment (TME) components. This poses questions about how reliably CCLs mimic patient tumours and the extent to which this represents an obstacle for the translation of CCL derived findings from‐bench‐to‐bedside.

If CCL characterizations, pharmacogenomic and genetic perturbation screenings are effective in the context of forward translation, which implies actualizing research discoveries into practice, reverse translation, that is the elucidation of the mechanistic basis of clinical observations, is a complementary and equally important need for successful drug development (Honkala *et al*, 2021). Hence, reverse translational practices such as the identification of clinically predictive features and their observational validation in real tumours is meaningless if it is not preceded by a correct selection of properly representative CCLs for each considered patient cohort.

In a precision medicine context, patients' genomic heterogeneity has been linked to differences in treatment response, and the efficacy of 75 FDA‐approved anti‐cancer drugs associated with 47 biomarkers across 25 cancer types (Feng *et al*, 2021). Indeed, efforts to genomically characterise tumour patients (International Cancer Genome Consortium *et al*, 2010; Cancer Genome Atlas Research Network *et al*, 2013) have also led to a comprehensive collection of data sets spanning across multiple omics, in some cases paired with clinical observations (Gao *et al*, 2013). This has allowed retrospectively validating to a certain extent some of the associations between molecular features and drug responses observed in CCLs. However, new CCL‐derived pharmacogenomic associations were not always confirmed in clinical trials. For example, the upregulation of IGFR1 found associated with tamoxifen resistance in breast cancer CCLs, exhibited the opposite behaviour in patients (Drury *et al*, 2011).

Leveraging the plethora of existing data, it is now possible to develop methods able to map CCLs to tumours, to identify CCLs that most closely resemble relevant patient characteristics to (1) achieve a better understanding of cancer mechanisms and (2) maximise the likelihood that virtual drug prescriptions discovered from CCL‐based studies are effective and beneficial for a specific patient segment.

Here, we first review the factors that might compromise how well CCLs represent primary tumours. We then discuss computational studies that investigate CCL resemblance to patient tumours, ranging from cancer‐specific investigations focussed on individual (or few) data modalities to more recent multi‐omic and pan‐cancer approaches. Finally, we offer an outlook on the use of machine learning methods in this context.

## Factors that might compromise how well cell lines represent tumour characteristics

**Figure 2:**
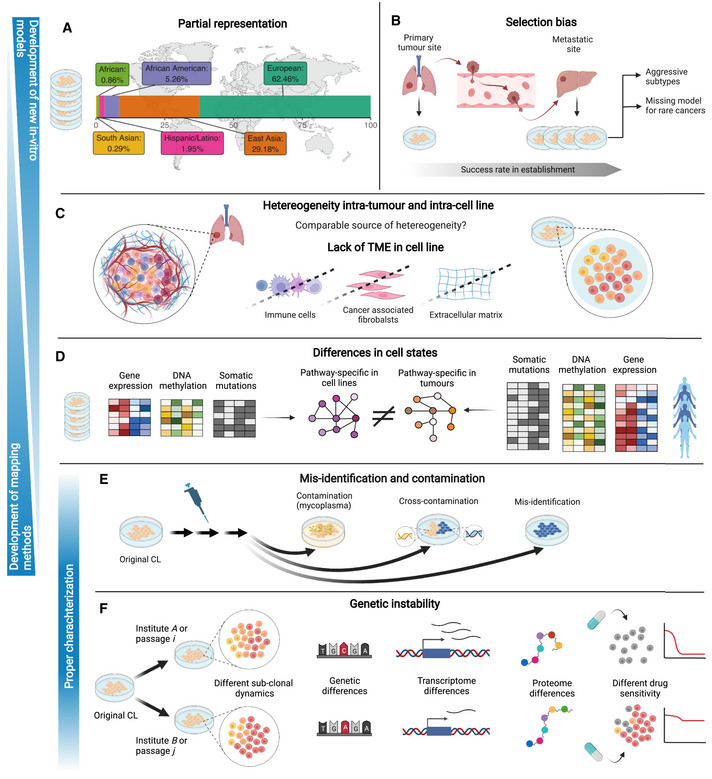
Factors hampering the faithfulness of CCLs as tumour models Panels A to E show issues that can be addressed by establishing new *in vitro* models (top to bottom) or by developing cell line‐tumour mapping methods (bottom to top). (A) Cell line biobanks are mostly derived from European and east Asian ancestries (data from Dutil *et al*, 2019). (B) Ease in establishing cell lines from more aggressive subtypes. (C) Intra‐tumour and intra‐cell lines dynamics, possibly reduced heterogeneity in cell lines that additionally do not include tumour microenvironment. (D) Differences in cell states among cell lines and tumour biobanks in terms of genetic, transcriptional, epigenomic and proteomic features that lead to differentially regulated pathways. (E) Contamination and mis‐identification due to lab conditions. Cells in blue represent a different donor. (F) Genetic instability in the same cell line due to different culture conditions or passaging can lead to divergences in genetic features, transcriptional and proteomic states and consequentially drug response. Created with BioRender.com.

The relevance of the findings originating from CCL‐based studies and their translation in clinical applications have been long questioned, long before large‐scale screenings became widespread (Hughes *et al*, 2007; Gillet *et al*, 2013). This was due to several factors that potentially compromise the faithfulness of CCLs in representing the cancer patients they are intended to model (Fig 2).

### Misidentification and contamination

The first issue arises from possible contaminations and misclassifications due to culturing the cells in the laboratory (Fig 2E). Cross‐contamination, for example cells from a foreign culture introduced accidentally in a CCL, is a well‐documented problem. Capes‐Davis *et al* (2010) performed a literature screening and reported that 360 CCLs from 68 references were cross‐contaminated, mostly intra‐species (90%), with the most common contaminant being HeLa cells (29%). Cross‐contamination cases are usually spotted by short tandem repeat (STR) profiling, generally performed for CCL authentication purposes, thus also suitable for the identification of mislabelled CCLs. Via STR profiling performed on 113 independent sources in China, 95 CCLs of the 380 tested were detected as cross‐contaminated, with 93.22% of the cases involving HeLa cells contamination (Ye *et al*, 2015). Another well‐known source of contamination is mycoplasma, a small parasitic bacterium which might be passed on by other contaminated cell cultures or laboratory personnel. RNA sequencing showed that 11% of 9,395 samples from hundreds of laboratories were indeed contaminated with mycoplasma (Olarerin‐George & Hogenesch, 2015).

Misidentification and misclassification errors arise when the gender, species, tissue or cell type, disease or CCL names are wrongly annotated and do not match the actual source. It was estimated that more than 30,000 scientific publications were affected by CCLs not being of the declared type (Horbach & Halffman, 2017). To tackle this significant problem, Cellosaurus (Bairoch, 2018) offers a CCL authentication system by STR profiling in the CLASTR (Cellosaurus STR similarity search) tool (Robin *et al*, 2020), which allows contrasting one or more STR profiles against that of 6,474 human CCLs. Despite being the international reference system for *in vitro* model authentication, STR profiling is still susceptible to heterogeneity within the same CCL that can occur due to differences in laboratory and culture conditions or genetic drifting, especially in the presence of microsatellite instability (Much *et al*, 2014).

### Genetic drift, *in vitro* selection pressures and genetic instability

Genetic heterogeneity in CCLs of the same origin has been observed by several groups (Ben‐David *et al*, 2018; Liu *et al*, 2019b; Quevedo *et al*, 2020), aggravating differences in models that originated from the same donor (Fig 2F). For instance, Ben‐David *et al* (2018) reported on many CCLs exhibiting complex clonal dynamics and evolutionary pressures specific to *in vitro* culturing conditions. Ben‐David *et al* (2018) reported that this in turn impacted functional properties such as morphology, proliferative capacity, gene expression and drug response. A recent study from Quevedo *et al* (2020) explored genetic stability across the 3 largest pharmacogenomic studies that leveraged both RNA‐seq and SNP array data, finding discrepancies both intra‐ and inter‐institutions, and hinting that pharmacological delineation could have been derived at different passages and/or stocks, thus not properly defining CCLs' drug responses and being linked to different and variable transcriptional programmes. Indeed, within‐CCL genetic variability greatly impacts gene expression, even at the level of cancer‐related genes (Fasterius & Al‐Khalili Szigyarto, 2018). Genomic and transcriptomic variations can translate also into variations at the proteomic level, as it was shown while investigating 14 HeLa cells strains (Liu *et al*, 2019b). On the contrary, epigenetic changes driven by environmental factors (e.g. culture conditions) are also plausible, given the evidence of DNA methylation instability in human pluripotent stem cells (Weissbein *et al*, 2017). However, no study has analysed this aspect so far. In addition, the mutational variability in a CCL donor can lead to the continuous emergence of new subclones in that CCL (Ben‐David *et al*, 2018), which can lead to the emergence of drug resistance (Hata *et al*, 2016).

In general, given the intrinsic differences between human physiological environment and cell culture conditions, it sems rather unlikely that tumours and CCLs are subjected to the same evolutionary selective pressures. This increases the molecular divergence between cancer models and the tumours they were originated from (reviewed in Ben‐David *et al*, 2019).

The similarity of CCLs to their tumour of origin can be considered a non‐critical issue if CCLs are used just as a means for investigating intrinsic oncogenic mechanisms. However, diverse evolutionary mechanisms could contribute to significantly distancing cancer model populations from patient cohorts, not properly mimicking the focal aspects of oncogenic addiction in cancer patients and their triggered genetic dependencies. This can lead to misleading findings that would not be recapitulated in real tumours. On the contrary, unstable molecular features in an *in vitro* cohort also limit the faithfulness of its molecular characterisation, making collected data inaccurate across multiple strains, with possible false findings arising especially when using CCLs for harvesting biomarkers of drug response.

### Selection bias

Existing biobanks and panels of *in vitro* cancer models are biased towards the preferential representation of certain cancer types and subtypes (Fig 2B). It has been reported that CCLs are more commonly derived from metastatic tumours due to their predisposition to grow successfully *in vitro* (Masters, 2000). The genetic changes accumulated by aggressive cancers are one possible explanation for their increased chances of growing indefinitely *in vitro*. Consequently, aggressive cancer subtypes tend to be overrepresented across CCL collections of a specific tissue. For example, breast CCLs are mostly derived from metastases rather than primary lesions (Burdall *et al*, 2003). In addition, CCL cohorts do not sufficiently represent all patient tumour subtypes (van Staveren *et al*, 2009; Klijn *et al*, 2015). This is a prominent problem for rare cancers which collectively make up 25% of cancer diagnoses each year (Greenlee *et al*, 2010) and for most of which no representative CCLs are available to date (Sharifnia *et al*, 2017).

### Missing tumour microenvironment factors

CCLs are cultured in flat plastic dishes, fed with synthetic media enriched with bovine serum and they completely lack the tumour microenvironment (TME) that surrounds patients' cancer cells *in vivo* (Fig 2C). The TME includes non‐malignant cell types such as immune cells and fibroblasts, extracellular matrix and signalling proteins (Binnewies *et al*, 2018). The crosstalk between tumour cells and the surrounding TME enhances both tumorigenesis and tumour progression, and also plays a role in preventing therapy efficacy and increasing multidrug resistance (Klemm & Joyce, 2015; Baghban *et al*, 2020). Furthermore, recent studies have shown that cell culture media impacts genetic dependencies observed in CCLs (Cheteh *et al*, 2017; Li *et al*, 2019; Rossiter *et al*, 2021). Nevertheless, despite the lack of immune‐like cells or cancer fibroblasts, it was found that specific metabolites in human plasma‐like medium also influence the set of essential genes in CCLs detected in CRISPR‐based screens (Rossiter *et al*, 2021). Co‐culturing CCLs with cancer‐associated fibroblasts (CAF) or even CAF‐conditioned medium reduced response to chemotherapeutic treatments and conversely increased cell survival in prostate CCLs (Cheteh *et al*, 2017). This offers the possibility to reproduce *in vitro* some of the interactions occurring between cancer cells and the TME. More complete TME representations have been implemented via co‐culturing technologies in complex *in vitro* models. For instance, three‐dimensional patient‐derived organoids (PDO) have been co‐cultured with endogenous native infiltrating immune cell populations and non‐immune stromal elements, allowing *in vitro* immune oncology investigations (Neal *et al*, 2018). In addition, single‐cell analyses in PDOs from pancreatic cancers showed that TME signals drive malignant cell states and influence drug responses (Raghavan *et al*, 2021). Interestingly, Raghavan *et al* (2021) also demonstrated that *ex vivo* soluble micro‐environment can be manipulated to alter transcriptional states, demonstrating again that at least some TME components can be modelled *in vitro*.

### Heterogeneity in tumours and cell lines

Individual patient tumours are typically highly heterogeneous in terms of their genetic, epigenetic, transcriptional, cell state and other phenotypic features (Marusyk *et al*, 2012; Jamal‐Hanjani *et al*, 2015). These different levels of intra‐tumour heterogeneity can arise from genetic instability followed by subclonal evolution, as well as epigenetic plasticity, diverse microenvironmental factors, and heterotypic interactions with immune and stromal cells (Hinohara & Polyak, 2019; Vitale *et al*, 2021). Recent work suggests that distinct genetic and molecular subtypes can often co‐exist within the same tumour (Patel *et al*, 2014; Roerink *et al*, 2018; preprint: Gavish *et al*, 2021; Raghavan *et al*, 2021). Such intra‐tumour heterogeneity plays a role in governing cancer progression and metastasis, as well as therapeutic response and resistance (Roider *et al*, 2020; Hong *et al*, 2019; Kim *et al*, 2018).

CCLs are typically believed to lack much of the representative heterogeneity of tumour cell populations due to the aforementioned *in vitro* culture conditions, lack of TME complexity and strong selective pressures induced by *in vitro* culturing that are thought to limit subclonal diversity. However, recent studies have casted doubt on the notion that CCLs models are made of homogenous, stable and clonal cell populations (Fig 2C).

Genetic heterogeneity in CCL models has been observed by different groups. For example, Ben‐David *et al* (2018) found that even single‐cell clones rapidly produce heterogeneous populations due to genetic instability. Minussi *et al* (2021) used single‐cell DNA‐Seq to characterise subclonal diversity in triple negative breast cancer and found that CCLs showed similar levels of subclonal diversity as tumours, and that this re‐emerged rapidly after single‐cell cloning. Similar subclonal dynamics have been observed to drive drug resistance in CCLs (Bhang *et al*, 2015). Single‐cell studies suggest that apart from genetic heterogeneity, CCL populations may additionally exhibit transcriptional heterogeneity, but not to the same extent as tumours. For example, recent pan‐cancer efforts aiming at characterising recurrent patterns of transcriptional heterogeneity in CCL models and tumours found that many of the transcriptional programmes driving intra‐tumour heterogeneity in patients were also observed in CCLs (Kinker *et al*, 2020; preprint: Gavish *et al*, 2021). Tumours exhibit significant heterogeneity also at the epigenetic level (Brocks *et al*, 2014). However, CCLs are largely underexplored at the epigenome level, and it remains to be determined how much of the transcriptional diversity observed in CCLs is rooted in their epigenetic heterogeneity.

Despite their complex and dynamic nature, CCLs unavoidably lack much of the tumour spatial organisation, cellular architecture and microenvironmental factors, and further understanding these similarities and differences in CCLs and tumours remains a key challenge. Homogenous *in vitro* models might be desirable for experimental studies of defined cancer types and states, as they allow pinpointing specific intrinsic molecular features. On the contrary, populations lacking representative sources of heterogeneity would fail to capture key aspects of patient tumours biology, dynamics and treatment response.

### Differences in genomic and cell state

Comparisons of tumours and CCLs, at bulk or single‐cell level, have indicated discrepancies with respect to somatic mutations and copy number alteration (CNA) frequencies, transcriptional and epigenetic states, (Fig 2D). Bulk gene expression profiling has identified pathway‐specific differences (Sandberg & Ernberg, 2005; Ertel *et al*, 2006). Pathways upregulated in CCLs are generally involved in metabolic processes, including cell nucleotide metabolism and oxidative phosphorylation, whereas downregulated ones typically involve cell adhesion and communication. Based on SAGE (Serial Analysis of Gene Expression) technology (Stein *et al*, 2004), 62 genes selectively overexpressed in tumours were found to be enriched for immune response and complement pathways, reflecting the presence of stromal and immune components, as well as extracellular matrix proteins. On the contrary, protein synthesis pathways were found dominantly enriched among the 61 genes overexpressed in CCLs. In addition, 5′C‐phosphate‐G‐3′ (CpG) islands were found more hypermethylated in CCLs, with more than 57% of model‐specific hypermethylated loci not being found in primary tumours (Smiraglia *et al*, 2001).

When considering transcriptional components involved in multidrug resistance (MDR), CCLs were observed to exhibit upregulation of genes that would facilitate survival (Gillet *et al*, 2011). This implies that CCLs are selected during their establishment via the expression of genes that are connected to MDR most likely as a response to environmental adversity. In addition, CCLs have been reported to be more sensitive to cytotoxic drugs compared with solid tumours, possibly due to their faster proliferation rate and their lack of a TME, which has been found to reduce responsiveness to chemotherapeutics (Marin *et al*, 2008; Straussman *et al*, 2012).

At the genome level, genetic aberrations characterising primary tumour are generally preserved in tissue‐matching CCLs. However, CCLs also tend to acquire novel locus‐specific alterations, several of which are rarely or never observed in primary tumours (Greshock *et al*, 2007; Tsuji *et al*, 2010), and show a generally higher frequency of mutations (Domcke *et al*, 2013; Jiang *et al*, 2016).

Considering the intrinsic differences between CCLs and primary tumours, Iorio *et al* (2016) focussed on multi‐omic cancer functional events (CFEs), that is molecular features derived by processing more than 11,000 primary tumour samples across 29 tissues with state‐of‐the‐art software aiming at identifying cancer driver alterations (Gonzalez‐Perez *et al*, 2013; van Dyk *et al*, 2013). The CFEs encompassed somatic mutations in cancer genes (CGs), recurrently aberrant copy number segments (RACs) involving at least a gene and affected in at least 2.5% of subjects, and hypermethylated informative CpG sites (iCpGs) in gene promoters with consistent hypo‐/hyper‐methylation. The status of the identified CFEs was then observed in more than 1,000 CCLs. Interestingly, this revealed that all pan‐cancer RACs identified in patients occurred in at least one CCL, followed by 89% of iCpGs and 64% of CGs. However, the correlation between CFEs occurrences in CCLs and patient tumours was high on average but highly variable across cancer types.

### Partial ancestry representation

Because available biobanks do not properly cover all ethnicities, CCLs are not representative of diverse ancestry (Fig 2A). This issue was clearly shown by Dutil *et al* (2019) using an interactive tool called ECLA (Estimated Cell Line Ancestry) that visualised ancestry of CCLs inferred from genome‐wide SNP array in the context of the 1,000 Genome project reference populations. Among more than 1,000 CCLs in CCLE and the COSMIC panels, European and East Asian account for 91.64% of the CCL ancestry. Moreover, 64 CLLs involve a discordant annotation between the genetically inferred ancestry and the self‐reported one.

This is a quite significant challenge considering that genetic variants associated with cancer risk could have a different effect across populations. For instance, variants detected in one population from genome‐wide association studies are not always recapitulated in a different ancestry or even display a different direction of association (Wang *et al*, 2018). One example is the rs2046210 variant at 6q25.1 in breast cancer, which is detected in asian and european women but not African‐American (Cai *et al*, 2011). A complete ancestry representation in *in vitro* models is essential for understanding how ethnicity differences impact cancer biology and to gain a comprehensive view of the underlying mechanisms.

## Computational methods for comparing cell lines and primary tumours

Some of the limitations hampering a correct modelling of primary tumours by CCLs can be circumvented computationally, by *in silico* preprocessing the related data. Particularly, appropriately mapping representative CCLs onto specific tumour segments can be achieved in a genomically guided fashion most preferably considering reprofiling CCLs immediately before possible screens for genetic dependency/drug sensitivity. This way it is possible (1) to elucidate at least the biological mechanisms that are retained in CCLs and (2) to facilitate translating CCL‐derived findings (for example from genetic dependencies and drug responses) into potential treatments for the mapped patient cohorts. A correct CCL‐to‐tumour matching overcomes CCL “misidentification” issues, reduces the effects of different culture conditions and allows focussing on features that are relevant to primary tumours while putting less emphasis on CCL‐specific ones.

### Interconnected objectives: integration, scoring and selection

**Figure 3:**
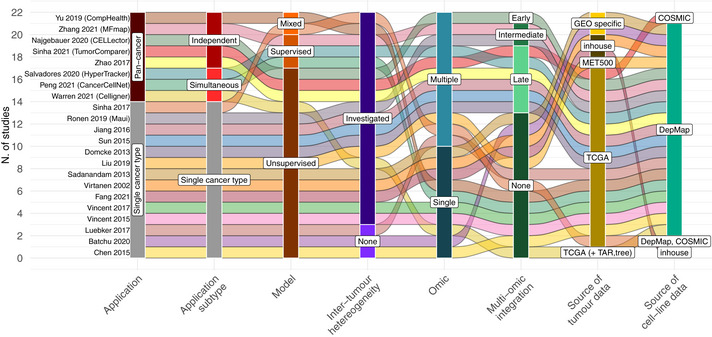
Number of studies classified based on the characteristic displayed on the x‐axis Each spline (alluvium) corresponds to a study in Table 2. “TAR” and “tree” abbreviations refer to TARGET and treehouse data set, respectively.

**Figure 4:**
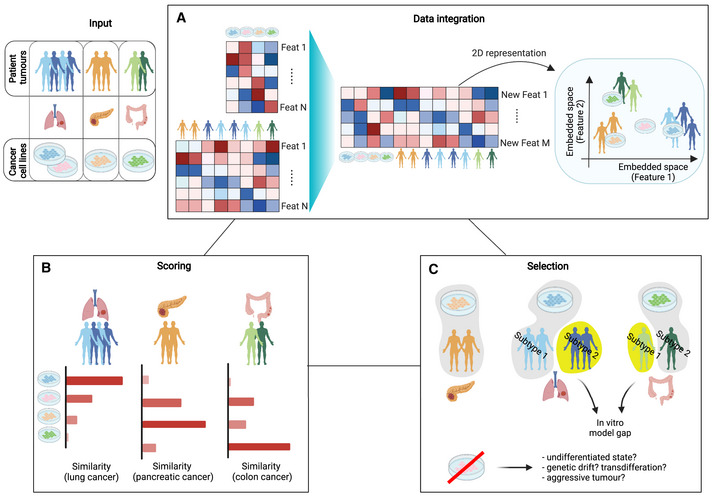
Aims of the major computational approaches proposed so far (A) Integration of cell lines and tumour in a common, comparable and visualisable feature space. (B) Scoring of cancer cell lines (CCLs) in terms of suitability in modelling a certain tumour population. (C) Selection of CCLs as proper model for tumour type/subtypes. Pursuing this objective can also highlight tumour populations lacking representative *in vitro* models and CCLs that diverge extensively from all the considered tumour populations. Created with BioRender.com.

Here, we review 22 studies that have leveraged multi‐omic data to compare the molecular characterisation of tumours and that of commercially available CCLs (Table 2 and Fig 3). To this end, we have classified these approaches based on three different but not mutually exclusive pursued objectives (Table 2 and Fig 4):

- ***integration*** of cell lines and tumour data.
- ***scoring***_,_ that is estimating a score to rank the quality of cell lines as tumour models.
- assignment and ***selection*** of cell lines as representative models for defined tumour type/subtype, with a consequent identification of gaps, that is tumours lacking representative cell lines.

**Table 2:** Methods/studies that map cell lines to tumours.

Reference	Data Input	Multi‐omic integration	Application	Unsupervised/Supervised	Clustering/subtype	Method		
						CCL—Tumour integration	Scoring CCL	Selecting CCL
Warren *et al* (2021) (Celligner)	GE	–	Pan‐cancer	Unsupervised	Subtype	Contrastive PCA + Mutual Nearest Neighbour	Pearson corr. on aligned space	–
						Assignment by k‐NN		
Peng *et al* (2021) (CancerCellNet)	GE	–	Pan‐cancer	Supervised	Subtype	–	Classification score	Multi‐class Random Forest
								“Correct” class: classification score > thr in actual type
Sinha *et al* (2021) (TumorComparer)	GE, CNA, Mut	Late	Pan‐cancer (independent)	Unsupervised	Subtype	–	Aggregated ranking of weighted Pearson's corr./Jaccard Index	–
Zhang & Kschischo (2021) (MFmap)	GE, CNA, Mut	Intermediate	Pan‐cancer (independent)	Supervised (subtype)	Subtype	ComBat (GE)	Cosine coefficient (latent space)	Neural network classifier on latent space
						Concatenated VAE		
Fang *et al* (2021)	PE	–	Thyroid Carcinoma	Unsupervised	Subtype	–	Pearson's corr.	–
Najgebauer *et al* (2020) (CELLector)	CNA, Mut, HypMet	Early	Pan‐cancer (independent)	Unsupervised	Clustering	–	Signature length times fraction of samples in group	Eclat clustering
								Map by decision tree
Salvadores *et al* (2020) (HyperTracker)	GE, HypMet	Late	Pan‐cancer	Supervised	Subtype	ComBat	–	Binomial ridge regression
								“Golden set” from matching data modalities
Batchu *et al* (2020)	GE	–	Alveolar Rhabdomyosarcoma	Unsupervised	–	–	Spearman's corr.	–
Yu *et al* (2019) (CompHealth)	GE	–	Pan‐cancer (independent)	Unsupervised	Subtype	ComBat	Spearman's corr.	TCGA‐110‐CL panel: 5 highest score per type
				Supervised (subtype)				
Liu, *et al* (2019a)	GE, CNA, Mut	–	Metastatic Breast Cancer	Unsupervised	Subtype	–	Spearman's corr. (GE and CNA)	–
Ronen *et al* (2019) (Maui)	GE, CNA, Mut	Intermediate	Colorectal cancer	Unsupervised	Clustering	Multimodal stacked VAE	Euclidean distance (latent space)	K‐means clustering (latent space)
								at least 1 of 5 NN being tumour
Zhao *et al* (2017)	GE, CNA, Mut	Late	Pan‐cancer (independent)	Unsupervised	Subtype	Distance weighted discrimination	Kendall Rank corr. (GE and CNA)	Similarity in at least 3 out of 4 modalities
							Gene Ontology enrichment score	
							Mutation presence	
Luebker *et al* (2017)	CNA, Mut	–	Melanoma	Unsupervised	–	–	Fraction of genome altered	–
							Pearson's corr. (CN)	
Vincent & Postovit (2017)	GE	–	Melanoma	Unsupervised	Subtype	–	Pearson's corr.	–
Sinha *et al* (2017)	GE, CNA, Mut	–	Renal Cancer	Unsupervised	Clustering/Subtype	ComBat	–	Hierarchical clustering (Spearman corr., CN)
				Supervised (subtype)				PAMR classifier (Spearman corr., GE)
Jiang *et al* (2016)	GE, CNA, Mut, PE	Late	Breast Cancer	Unsupervised	Clustering/Subtype	–	Sum Pearson corr.	Hierarchical clustering (PE, GE)
Sun & Liu (2015)	GE, CNA	Late	Breast Cancer	Unsupervised	Subtype	–	Aggregated ranking of Spearman's corr.	–
Vincent *et al* (2015)	GE	–	Breast Cancer	Unsupervised	Subtype	‐	Pearson's corr. (group specific)	‐
Chen *et al* (2015)	GE	–	Hepatocellar Carcinoma	Unsupervised	–	–	Spearman corr.	–
Sadanandam *et al* (2013)	GE	–	Colorectal cancer	Unsupervised	Clustering	Distance weighted discrimination	–	SAM and PAM for feature extraction
								Consensus‐based NMF
Domcke *et al* (2013)	GE, CNA, Mut	Late	Ovarian Cancer	Unsupervised	Subtype	‐	sum: CNA Pearson corr. and Mut presence/absence	GE for validation: hierarchical clustering
Virtanen *et al* (2002)	GE	–	Lung Cancer	Unsupervised	Clustering	Lowess normalisation	–	Hierarchical clustering
								Comparison with known label

CCL, cancer cell line; CNA, copy number alterations; GE, gene expression; HypMetm DNA methylation; Mut, somatic mutations; PE, protein expression.

While most of the studies (14 of 22) focus on a single cancer type, more recent ones are applied to a pan‐cancer context (8 of 22). Among those, only 3 describe methods analysing all cancer types with available data jointly, while the remaining ones are separately applied to individual cancer types (*Pan‐cancer independent)*. Inter‐tumour heterogeneity within the same cancer type is accounted for in 19 of 22 studies either using prior known subtype labels (for example, consensus molecular subtypes) or determined in a data‐driven way via cluster analysis. In general, when comparing CCLs and tumours, the preferred approach is unsupervised (17 of 22), estimating global similarities between samples on a domain of informative features only. Instead, 3 of 22 methods adopt a supervised approach, building predictive models which are trained on primary tumours' data to capture a phenotype of interest (tumour type/subtype), then used to classify CCLs. Finally, 2 studies include both strategies, unsupervised for general CL‐tumour comparison and supervised for tumour subtype assignment.

Large‐scale publicly available databases, such the CCLE and those collected under the DepMap portals, are typically used as the source of CCL data (19 studies), plus an earlier application (Virtanen *et al*, 2002) used data related to CCLs that were subsequently collected in DepMap. Seventeen studies use TCGA as a source of primary tumours' data. Moreover, despite the developed methods not being strictly subordinate to the data type included (gene expression, somatic mutations, copy number alterations, DNA methylation and/or protein expression), we observed heterogenous choices of used omic(s). More importantly, only 9 of 22 studies address multi‐omic sources with disparate strategies for their integration. Here, we only focus on studies comparing tumours to CCLs, but we nevertheless note that 2 of the listed publications (Liu *et al*, 2019b; Peng *et al*, 2021) extend their methods to more complex models such as tumoroids and PDx, highlighting different representative performances and quality across model complexities.

Only 9 studies apply a data integration method that goes beyond a straightforward juxtaposition of the two data sources (CCLs and primary tumours) based on a common and/or most variable set of features and performing a data harmonisation in an integrated comparable space (Fig 4A and Table 2). This is usually implemented when handling gene expression data, via multi‐platform microarray integration methods (for example, distance weighted discrimination and lowess normalisation), or borrowing techniques for experimental batch correction, such as ComBat (Johnson *et al*, 2007) or mutual nearest neighbour (MNN) (Haghverdi *et al*, 2018). The benefits of data integration are clearly shown in (Salvadores *et al*, 2020; Warren *et al*, 2021), which display a 2D projection of combined CCLs' and primary tumours' data in an uncorrected and corrected version (i.e. contrastive PCA followed by MNN or quantile normalisation plus ComBat), with only the second case showing the two datasets properly mixed, while maintaining tissue type separations. A different approach is applied in (Ronen *et al*, 2019; Zhang & Kschischo, 2021), based on a variational autoencoder (VAE) that identifies, in an unsupervised manner, non‐linear latent factors from the initial feature space common to both CCLs and tumours.

The scoring objective is instead one of the most pursued across the examined methods (Fig 4B and Table 2). This goal is usually achieved through the use of a correlation (Spearman's or Pearson's) or similar metric (Kendall or Jaccard index, Euclidean distance or cosine coefficient) (Domcke *et al*, 2013; Chen *et al*, 2015; Sun & Liu, 2015; Vincent *et al*, 2015; Jiang *et al*, 2016; Luebker *et al*, 2017; Sinha *et al*, 2017, 2021; Vincent & Postovit, 2017; Liu *et al*, 2019a; Ronen *et al*, 2019; Batchu *et al*, 2020; Fang *et al*, 2021; Zhang & Kschischo, 2021), sometimes applied to a new “corrected” feature space (Warren *et al*, 2021). This similarity score is usually computed first sample‐wise, then for each CCL averaged across tumours from a given tumour type/subtype (usually matching that in the CCL annotation). The approaches that work this way build on unsupervised strategies that focus on a global similarity metric. Supervised methods, on the contrary, first build a classification model that can learn discriminative features between tumour types/subtypes and then examine and classify CCLs based on the status of these features. Although more appropriate for the selection tasks, classification “scores” can also be used as a quality estimation and hence to rank CCLs based on their representative quality (Peng *et al*, 2021). Regardless of whether a certain classification is correct, studies including a scoring objective can indicate the most fitting CCL for every tumour‐type/subtype under investigation.

With respect to the selection objective (Fig 4C and Table 2), not all methods proposed so far clearly pinpoint a set of CCLs as representative models for the tumour type under investigation. Although selection could be a consequence of scoring via the application of a filtering threshold on the estimated scores (Yu *et al*, 2019), this is usually not the case from correlation‐based studies. Instead, supervised methods more naturally assign a CCL to a tumour of interest and a representative set of CCLs is then obtained as those retaining their tissue identity following the classification (Salvadores *et al*, 2020; Peng *et al*, 2021).

One of unsupervised methods, CELLector (Najgebauer *et al*, 2020), initially clusters primary tumours in an unsupervised way based on the most co‐occurring combinations of genomic features (signatures) and subsequently assigns CCLs to a patient segment conditional to the presence of the underlying signature, without relying on a similarity score. Although a score is built as a product of the percentage of the tumour samples covered by a patient segment and the granularity of the underlying signature (in terms of number of accounted features), this is more representative of the patient subgroup “quality” and informativeness rather than the CCL ability to represent that patient cohort.

Generally, pursuing the selection objective helps also identifying tumour types and subtypes lacking adequate model representation, providing guidelines for new *in vitro* model development. Moreover, it boosts pharmacogenomic associations whose significance is diluted when accounting for low‐quality models (Najgebauer *et al*, 2020; Salvadores *et al*, 2020; Peng *et al*, 2021; Zhang & Kschischo, 2021). For instance, (Salvadores *et al*, 2020) identified drug sensitivity markers across cancer types using only “golden sets” of CCLs strongly resembling their cancer type of origin based on transcriptomic and epigenomic profiles. They found a higher number of pharmacogenomic associations across tumour types compared to using data from all available CCLs, including a previously unreported association between CDKN2A losses and camptothecin sensitivity in glioblastoma. On the contrary, removing non‐representative/low‐quality CCLs from pharmacogenomic associations studies filters out likely not relevant statistical interactions between drug responses and genomic features. For instance, in colorectal CCLs, BRAF mutations are no longer associated with dabrafenib responsiveness, consistent with what was observed in tumour patients. Najgebauer *et al*, 2020 used a different approach, where CCLs were grouped according to the genomic signatures underlying the patient segments they were mapped onto, and assessed for differential drug response across the resulting stratification. This yielded 88 unique drugs whose differential response is more significantly associated with a signature of genomic alterations than it is to individual genomic events. As an example of precision medicine application, this approach also shows that refining the subpopulation of KRAS mutated lung adenocarcinoma CCLs based on 2 complementary signatures (TP53 mutant and ARL17A promoter hypermethylation and the absence of those plus the absence of GSTT1 promoter hypermethylation, respectively) increases the differential drug response significance for the MEK1/2 inhibitor selumetinib and the BRAF inhibitor dabrafanib. Finally, scoring/selection objectives can identify cell state discrepancies between tumours and CCLs, as well as spot mis‐identified CCL clearly exhibiting features of a different tumour type, allowing for their reclassification in a pan‐cancer context.

### Pan‐cancer approaches identify discrepancies in cell lines states

Among the 22 studies considered here, only 3 develop a pan‐cancer approach that simultaneously considers all CCLs and tumour types with available data (Salvadores *et al*, 2020; Peng *et al*, 2021; Warren *et al*, 2021), all focussing on transcriptional states and data. HyperTracker (Salvadores *et al*, 2020) and CancerCellNet (Peng *et al*, 2021) adopt a supervised approach to build a cancer type classifier using primary tumour data for training based on a *one‐versus‐rest* binomial ridge regression and a multiclass random forest, respectively. Cancer type labels are then predicted by the trained classifier for the CCLs and compared with tissue/cancer‐type labels they were originally annotated with. This allows to partition the analysed CCLs into (1) correctly classified (“golden/silver” set or “correct” class), (2) high confidently predicted to be derived from a cancer type different from the annotated one (“suspected” set or “other” class) and (3) ambiguously assigned to more than one cancer type, with low certainty or no concordance among multiple data types (“undetermined” set or “none/mixed” class). On the contrary, Celligner (Warren *et al*, 2021) uses an unsupervised approach, creating a “corrected” gene expression space through the simultaneous integration of CCLs and primary tumours across cancer types, hence allowing to detect distinct subpopulations and cross‐cancer‐types affinities. For each CCL, a comparison between its 25‐nearest tumour samples labels in the new space and the original annotation enables identifying differences in CCLs states. Only portions of analysed CCLs retain the original label state as correctly predicted across the considered studies (60% in (Salvadores *et al*, 2020; Warren *et al*, 2021)) and with variable results across cancer types. For instance, in (Peng *et al*, 2021), a CancerCellNet application shows that in only 6 of 20 cancer types more than 50% of CCLs assignment match their original label. Cancer types with poorly aligned CCLs are those that originated from pancreas, thyroid, oesophagus and central nervous system (CNS) tissues, consistently across studies.

Historically, glioblastoma (GBM) cell models exhibit a distinct transcriptional state than primary tumours and tend to lose their ability to differentiate when grown in serum‐containing media (Lee *et al*, 2006; Ledur *et al*, 2017). Indeed, CancerCellNet assigns the majority of GBM CCLs to the sarcoma type, similarly to Celligner that places 82% of CNS CCLs as part of an undifferentiated/mesenchymal group. In addition, when training the classifier on single‐cell RNA‐seq data, CancerCellNet (Peng *et al*, 2021) classifies 25 of 31 GBM lines as GBM neoplastic cells with 10 lines ambiguously being assigned also to the cancer‐tumour fibroblast class, possibly due to a derivation from a mesenchymal subtypes. Of note, low‐grade glioma (LGG) is consistently underrepresented across studies in CCLs collections even when considering co‐occurence of genomic cancer‐functional events, with 95% of tumour patients lacking a representative *in vitro* model (Najgebauer *et al*, 2020). Moreover, 91% of thyroid CCLs are also part of the transcriptionally based undifferentiated group (Warren *et al*, 2021), consistent with previous findings (Pilli *et al*, 2009).

Although an undifferentiated state is not a specific characteristic of pancreatic and oesophageal CCLs, the majority of CCLs annotated as being derived from these tissues are not predicted as adequate tumour models and further reasons related to this, for example propensity to metaplastic events, should be investigated (Wang & Souza, 2011). In general, a pan‐cancer approach, especially based on transcriptional levels, allows the identification of a common undifferentiated state, possibly representative of known tumour subtype (e.g. dedifferentiated melanoma), due to artefacts from 2D culture or indicative of a stem‐like state or an aggressive tumour cell state which is not detectable from bulk tumour data.

Another advantage of pan‐cancer approaches is that they can properly reclassify *in vitro* models when multiple sources are considered. For instance, in (Salvadores *et al*, 2020), HyperTracker identified a set of 43 CCLs with transcriptomic and epigenomic profiles significantly different from those of their originally annotated cancer types. A closer inspection re‐classified 22 of these CCLs, based on similarities spanning multiple data modalities, to a different cancer type. The authors provide two possible explanations for CCLs with discordant predicted/annotated tissue labels: misidentifications at the time of isolation or transdifferentiation. As label reassignment is supported in this study by multiple independently generated data omics, the authors conclude that the 22 lines were most likely misidentified. This is because transdifferentiation, that is CCL divergence during cell culture towards another cancer type, would be inclined to strongly affect the transcriptome/epigenome while having a reduced impact on the genetic component.

TumorComparer (Sinha *et al*, 2021) considers a weighted correlation metric to compare tumours and CCLs, across individual omics separately. This metric weights more cancer relevant features and results into similarity scores that are subsequently aggregated across omics layers. In this way, the authors identify 69 outlier CCLs (not sufficiently similar to any other tumour type) that need to be further investigated to determine whether they are from an undifferentiated state, or they have been mislabelled or possibly subjected to other molecular divergences. Finally, non‐pan‐cancer studies could also identify CCLs with discordant predicted/annotated labels which can be repurposed for studies investigating a cancer tissue different from that they were thought to model originally. For instance, Chen *et al*, 2015 detected 7 non‐liver CCLs whose molecular profile significantly correlates with that of hepatocellular carcinomas, and 2 or them (the pancreatic CCL TCC‐PAN2 and the stomach CCL FU97) even exhibit a higher level of similarity than actual liver CCLs. These two CCLs could be therefore used in hepatocellular carcinoma studies with a focus on transcriptomic data. In conclusion, pan‐cancer approaches proposed so far leverage the genomic differences between the cancer types and can identify CCLs with discordant states from their supposed tumour of origin.

### Addressing inter‐tumour heterogeneity

Recent technological advances such as single‐cell sequencing are starting to shed light on intra‐tumour heterogeneity, that is genomic and physiological variations within a tumour gained by cell evolution under selective pressures and microenvironmentally driven epigenetic modulation (Jamal‐Hanjani *et al*, 2015; Hinohara & Polyak, 2019). Nevertheless, the prevailing understanding of patient tumour heterogeneity is still restricted to differences across patient genomic profiles, thus leading to disease subclassification. This inter‐tumour heterogeneity has been linked to differences in treatment response and used for the therapeutic management of different cancer types (Heiser *et al*, 2012; Ceccarelli *et al*, 2016; Liu *et al*, 2018). Consequently, an important task is to identify *in vitro* models most resembling a certain tumour molecular subtype to draw correct conclusions when examining drug efficacy and genetic dependencies, which might be specific to individual patient subcohorts. As an intrinsic characteristic of tumour cohorts, inter‐tumour heterogeneity is investigated in almost all studies that we have considered in this Review (Table 2 and Fig 3), either via unsupervised clustering of patient data (6 of 22) or leveraging their *a priori* defined molecular subclassification (15 of 22), sometimes at the CCL level (Domcke *et al*, 2013; Vincent *et al*, 2015; Fang *et al*, 2021). The strategies adopted to account for tumour heterogeneity are numerous and disparate. For instance, Celligner (Warren *et al*, 2021) examines the intra‐cluster variability in a corrected gene expression space that integrates CCLs and tumours, finding this reflective of known tumour subclassifications for breast, kidney, leukaemia and skin cancer. Conversely, supervised methods (Sinha *et al*, 2017; Yu *et al*, 2019; Salvadores *et al*, 2020; Peng *et al*, 2021; Zhang & Kschischo, 2021) use prior known subtype labels of patient tumours to build a classifier and then predict the subtype of CCLs, similarly to the strategy adopted in the pan‐cancer approaches described in the previous section. Unsupervised strategies based on a correlation metric ranked CCLs based on their average similarity to tumour subtypes (Sinha *et al*, 2021; Fang *et al*, 2021; Liu *et al*, 2019b; Vincent & Postovit, 2017). Molecular subtypes are still being established and refined to better capture disease progression, and prior known subtypes could be assigned through a human inspection, which is error prone. For that reason, it is still relevant to discover and integrate classification systems built on other genomic features. Indeed, two studies perform unsupervised patient clustering instead of relying on known partitions, Maui (Ronen *et al*, 2019) focussing on colorectal cancer and CELLector (Najgebauer *et al*, 2020) considering 16 cancer types independently. Maui (Ronen *et al*, 2019) applies a multimodal stacked variational autoencoder (VAE) to integrate CCLs and primary tumours in a latent space on which cluster analysis is then performed. Following this, the authors report that multi‐omic‐derived clustering is more powerful than transcriptionally derived consensus molecular subtyping (CMS (Guinney *et al*, 2015), widely used by the community) as it reveals distinct CNAs, mutation and methylation profiles not detected only based on the gene expression classification. In addition, Maui assigns CCLs to the closest group in a latent space that hence resembles these genomic changes. CELLector (Najgebauer *et al*, 2020) instead leverages clinical relevant genomic and epigenomic features (Iorio *et al*, 2016) and unveils inter‐tumour heterogeneity by partitioning patients samples based on the most frequently occurring sets of molecular signatures. CCLs are then assigned to patient subcohorts based on the collective presence or absence of these features.

The investigation of inter‐tumour heterogeneity also allows to detect patient subgroups lacking representative models, especially for strategies that include a selection objective. For example, Najgebauer *et al*, 2020 estimate that across cancer types and 14 TCGA cohorts (4,153 samples), 11.7% of patients belong to segments with no representative *in vitro* models in the CELLector search spaces built using CNAs and mutations in high confidence genes. This percentage varies across cancer types. In particular, LGG and prostate adenocarcinoma (PRAD) are the most underrepresented cancer types with 95% and 62% of patients without matching CCL models. Even for widely studied cancers such as LUAD, the large cohort of CCLs fail to represent 3% of patients characterised by mutation in KRAS and ATM and the absence of mutations in TP53 and STK1 genes. For annotated subtypes, Sinha *et al*, 2017 report that no kidney‐derived CCLs cluster with chromophobe renal cell carcinoma (RCC) (a more indolent and less prevalent subtype than other RCCs) based on CNAs. Accordingly, based on transcriptional data, the cluster of chromophobe RCC tumour samples does not incorporate any CCLs in the Celligner‐corrected space (Warren *et al*, 2021). Celligner also reveals that there is a underrepresentation of transitory melanoma subtype due to the fact that CCL derive from metastatic tumours rather than primary tumours. In a subtype classification implemented across 15 cancer types by HyperTracker (Salvadores *et al*, 2020), half of the tumour subtypes are not represented by any CCLs in kidney, bladder and brain cancer. In particular, 78% of GBM and LGG CCLs are assigned to a mesenchymal‐like type in agreement with other studies (Peng *et al*, 2021; Warren *et al*, 2021). To understand whether CancerCellNet classification was not successful when considering multiple cancer types (i.e. assigned to a “Mixed” or “None” prediction) due to the presence of a strong diverging subtype for a certain cancer type, Peng *et al*, 2021 performed a subtype classification for 11 cancer types, accounting also from subtypes defined from histology or molecular profiles. Interestingly, 25 CCLs (13% of the analysed cohort) without a successful classification in the general framework are in this case reliably classified as a specific subtype that hence exhibit features not shared across cancers from the same tissue. The CancerCellNet subtype classification also highlights the absence of representative CCLs for basal and secretory LUSC, terminal respiratory unit LUAD and indicates only one representative CCL for endometrioid carcinoma. These results are indicative of a selection bias towards deriving CCLs from aggressive tumour types. In a breast cancer analysis (Sun & Liu, 2015), a subset of CCLs shows low similarity to any of the breast tumour subgroups, most likely because they are derived from metastasis. Sinha *et al*, 2017 reported that kidney tumours clustering with kidney‐derived CCLs are representative of a more aggressive state, namely clear cell RCC. Vincent *et al*, 2015 show that breast CCLs of the more invasive basal subtype are transcriptionally more similar to their respective tumours than luminal CCLs.

Using a supervised approach based on nearest template prediction, Yu *et al*, 2019 built a gene expression‐based predictive model trained on primary tumour data and then inferred cancer subtype status in CCLs from nine cancer types. All subtypes had a predicted representative CCL; however, the proportions of representative CCLs across subtypes significantly differs in breast invasive carcinoma (BRCA), LUAD and skin cutaneous melanoma (SKCM). In particular, the predominant predicted classes for the CCLs are basal for BRCA, proximal inflammatory/proliferative for LUAD, keratin/mitf_low for SKCM, all corresponding to poor prognosis groups with medium‐to‐low survival rates. Finally, the subtype classification from HyperTracker presented in (Salvadores *et al*, 2020) finds a single predominant subtype predicted in CCL panels for liver, skin and thyroid cancers.

In conclusion, the selection bias in establishing *in vitro* models from more invasive cancer subtypes appears clearly from the inter‐tumour heterogeneity investigations of the reviewed methods, also highlighting many cases of cancer subtypes lacking representative *in vitro* models.

### Unveiling biases in current cell line usage

In 9 of the studies discussed in this Review, the authors determine the number of times individual CCLs are mentioned across manuscripts published in peer‐reviewed journals (Sinha *et al*, 2021; Liu *et al*, 2019b; Yu *et al*, 2019; Zhao *et al*, 2017; Sinha *et al*, 2017; Jiang *et al*, 2016; Vincent *et al*, 2015; Chen *et al*, 2015; Domcke *et al*, 2013). This reveals that the most frequently used CCLs are not usually those more genomically similar to tumours. The reasons for this usage bias could lie on the ease in obtaining specific CCLs, their growing efficiency or a mere literature miscommunication.

Domcke *et al*, 2013 were the first to highlight this controversy for ovarian cancer. Focussing on high‐grade serous ovarian cancer (HGSOC), the most prevalent subtype, they found that the most used CCLs, SK‐OV‐3 and A2780 accounting for 60% of the total HGSOC CCL citations in the analysed literature (3,464 studies), greatly diverge from patient tumours when comparing CNAs profiles and the absence/presence of subtype specific mutations (e.g. TP53 mutations). The authors also highlight 12 CCLs more genomically similar to primary tumours which are generally less considered, with at the time only 1% of PubMed citations, and whose selection should be prioritised when establishing a new *in vitro* study. These results are also confirmed in a later study (Zhao *et al*, 2017) that additionally includes gene expression and functional similarity across gene ontology terms, and ranked SK‐OV‐3 and A2780 poorly in representing ovarian primary tumour. Screening eight cancer types, Zhao *et al* (2017) find further inconsistencies in intestine adenocarcinoma, with HT‐29 being the most cited CCL (∼ 18,000 PubMed citations) but ranking only at the 30^th^ position based on molecular faithfulness to patient tumours. Similarly, the most used breast CCLs MCF‐7 and MDA‐MB‐231 (collectively accounting for > 53,000 PubMed citations) are also not high‐quality models of primary tumours, in contrast to the less cited T47D, SK‐BR‐3, MDA‐MB‐468 and BT483 CCLs. In line with this, Jiang *et al*, 2016 showed that the highly cited breast CCLs MCF‐7 and MDA‐MB‐231 only rank 17^th^ and 21^st^ as most similar to tumours based on a comparative correlation sum that combines gene expression, mutational profiles, CNAs and protein expression and instead assign to the less cited BT483 and T47D lines the highest similarity to tumour scores. MCF‐7 and MDA‐MB‐231 were also reported to be poorly representative of metastatic breast cancer by (Liu *et al*, 2019a), who for example reported MCF‐7 as reliably classifiable as of luminalB subtype. On the contrary, MDA‐MB‐231, which is used as a triple‐negative metastatic breast cancer model across many studies, could not be assigned to any subtype and ranked poorly in terms of correlation to basal‐like metastatic tumour samples. These findings were also independently confirmed using gene expression data (Vincent *et al*, 2015). This study who directly considered known CCL partitions in luminal/basal types and compared them with a similar classification of primary tumours, ranking MCF‐7 and T47D as 5^th^ and 6^th^ best models for luminal subtype and MDA‐MB‐231 in 17^th^ position for basal subtype. Furthermore, Yu *et al*, 2019 highlight that the most used CCL for pancreatic adenocarcinoma (PAAD), MIA PaCa‐2 (∼ 1,000 PubMed citations), is the least transcriptionally similar to primary tumours, across a panel of 41 pancreatic CCLs, likely due to neuroendocrine differentiation. Discrepancies also arise for highly cited CCLs subtype annotations compared with their genomic features. For instance, the IGROV1 CCL is often quoted as HGSOC, but it is found more fitting as a model for endometrioid or ovarian clear cell carcinoma due to co‐occurrence of PIK3CA and PTEN mutations and expression‐based clustering in (Domcke *et al*, 2013).

In a study on renal cell carcinoma (RCC) subtypes based on CNAs profiles, Sinha *et al* (2017) find that ACHN is the third most cited CCL with a generic RCC annotation. However, it specifically clusters with the less prevalent papillary subtype, covering only 15% of RCC tumours. Overall, application of TumorComparer (Sinha *et al*, 2021) across 24 cancer types finds 69 CCLs detected as outliers based on an aggregated correlation from gene expression, CNAs and somatic mutations to their tumour of origin, of which 31 exceedingly 1,000 PubMed citations. Although, in this study, CCLs could be categorised as outliers even close to less frequent subtypes, these results are still indicative of biases in *in vitro* model selection. Of note, this phenomenon is not always occurring. For example, HepG2, a widely used hepatocellar carcinoma CCL, is reported as the highest quality model based on transcriptional correlation with patient tumours in (Chen *et al*, 2015).

It is important to stress that the performed citation searches are agnostic with respect to the usage of the cited models in the considered studies. Hence, false positives could be present due to publications using a CCL as a generic validation tool rather than for investigating cancer type/subtype‐specific mechanisms. As an example, (Gonçalves *et al*, 2021) use the HT‐29 CCL just as a tool model for testing the performances of a new CRISPR‐Cas9 library of guide RNAs. Nevertheless, these studies show that there is a clear bias from a literature search in specific CCLs usage and this highlights the importance of assessing the suitability of a CCL as a proper model of the tumour under investigation at the early stages of an experiment, without being drawn towards the easiest to retrieve or to grow *in vitro* models.

### Challenges from tumour impurity

In constrast to *in vitro* models, tumours are surrounded by a TME composed of stromal, immune cells and extracellular matrix. Accounting for these factors while comparing tumours and CCLs is particularly challenging when using data derived from bulk experiments. Indeed, bulk experiments for profiling copy number alterations, gene expression and DNA methylation do not differentiate among malignant and non‐malignant cell types, rather giving a mixed view of all cells in the tumour sample. To understand the extent of malignant cell fraction in bulk data, computational methods estimating tumour purity have been developed (Carter *et al*, 2012; Yoshihara *et al*, 2013; Aran *et al*, 2015).

Although all the methods discussed in this Review analyse data from bulk experiments, tumour purity is investigated in 9 of 22 studies, mostly when considering gene expression (Vincent *et al*, 2015; Luebker *et al*, 2017; Vincent & Postovit, 2017; Liu *et al*, 2019b; Yu *et al*, 2019; Batchu *et al*, 2020; Salvadores *et al*, 2020; Peng *et al*, 2021; Warren *et al*, 2021). A frequent strategy is to exclude genes whose expression pattern across samples is found highly correlated with sample purity scores (or their surrogate), using an *a priori* decided filtering threshold (Vincent *et al*, 2015; Vincent & Postovit, 2017; Yu *et al*, 2019; Batchu *et al*, 2020). Yu *et al* (2019) show that this method alleviates a similarity bias: indeed, the elevated presence of stromal and immune cells decreases the similarity between tumours and CCLs. Nevertheless, the relationship between tumour sample purity and CCL‐tumour correlation became not significant if signatures of high‐impurity genes are removed from the comparison, and the expression patterns of the remaining ones are additionally corrected for purity scores. On the contrary, the contribution of the immune infiltrate component cannot be entirely removed, as it has been shown from differential analysis that the protein–protein interaction network of upregulated genes in primary tumour is still enriched for genes in the immune response pathway.

The same approach of removing genes whose expression pattern is highly correlated with impurity scores was applied in a study focussing on alveolar rhabdomyosarcoma (Batchu *et al*, 2020); however, this fails to alleviate differences between CCLs and primary tumour, as indicated by a principal component (PC) space inspection. Principal component analysis detects as major source of variability differences in TME in (Vincent *et al*, 2015; Vincent & Postovit, 2017). In particular, PCs computed on the juxtaposed CCL‐tumour gene expression data sets reveal a clear separation between the two data sources, with PC2 being correlated with lymphocyte density in melanoma (Vincent & Postovit, 2017) and PC1 with stromal scores in breast cancer (Vincent *et al*, 2015).

Furthermore, implementing filtering strategies aiming at limiting TME differences is not always performed via gene removal. In a melanoma study (Luebker *et al*, 2017), gene expression is used to estimate tumour cell fraction on a patient sample but then tumour samples with high tumour impurity are just removed from the analysis.

Without a prior knowledge of tumour purity but with a more sophisticated data integration step, Celligner (Warren *et al*, 2021) combines gene expression data for *in vitro* models and primary tumours in a multiple‐step procedure. First, a contrastive principal component analysis (cPCA) is applied to detect variability enriched in one data source with respect to the other and vice versa, and the first four cPCAs are removed. Then, mutual nearest neighbour (MNN) correction is applied using the CCL data as a reference. This ad hoc procedure highlights that tumour‐specific signatures associated with the first cPC are enriched in immune pathways and that the second cPC is highly correlated with tumour purity estimates. Despite accounting for the first four cPCs, TME effects still persist and are later captured by the MNN step.

Of note, two pan‐cancer‐supervised methods (Salvadores *et al*, 2020; Peng *et al*, 2021) do not focus on CCL‐tumour differences driven by normal cell contamination, but instead investigate whether tumour impurity interferes with model prediction accuracies. In particular, HyperTracker (Salvadores *et al*, 2020) integrates CCL and tumours via quantile normalisation plus a ComBat application (Leek *et al*, 2012), and CancerCellNet (Peng *et al*, 2021) converts gene expression matrices in binary gene‐pair formats, assigning 1 if the first gene in the pair has higher expression than the second gene within a sample. In both cases, the results show that purity does not affect the model estimates: HyperTracker AUPRC values are very similar when training on low or high purity TCGA samples, and CancerCellNet mean scores have only a marginal correlation with mean sample purity (correlation = 0.14).

Finally, single‐cell technologies provide a unique opportunity to clear up tumour infiltrating cells, allowing the comparison between CCLs and pure populations of malignant cells from a patient tumour (Vincent & Postovit, 2017; Peng *et al*, 2021). For instance, Vincent & Postovit (2017) show an improved correlation among CCLs and malignant cells from primary tumours in melanoma compared to accounting for all cell types (0.83 and 0.67 respectively).

In conclusion, while TME effects cannot be entirely removed from bulk experiments, a proper integration strategy can alleviate the immune and stromal related differences leading to more reliable cell‐lines versus tumours comparisons.

### Feature selection strategies

The quality of CCLs also depends on features and biological states considered as relevant when they are compared with primary tumours. Most of the studies reviewed here focus more on comprehensive comparisons, aiming at assessing CCLs resemblance to tumours across the largest possible number of available features. In this respect, Celligner (Warren *et al*, 2021) uses the top 1,000 genes with the highest inter‐cluster variance within each data type. After initially addressing tumour‐CCL variability via cPCA, this tool analyses the remaining highest sources of variation that could discriminate against cancer types. In contrast, other pan‐cancer methods (Yu *et al*, 2019; Salvadores *et al*, 2020; Peng *et al*, 2021) focus on most variable features across cancer types when analysing tumour data. For example, CancerCellNet (Peng *et al*, 2021) selects genes coming up as highly differentially expressed when contrasting a cancer type versus all other samples. Similarly, HyperTracker (Salvadores *et al*, 2020) and CompHealth (Yu *et al*, 2019) select the 5,000 genes with the most variable expression pattern across all tumour samples. This type of filtering prioritises features that are discriminatory across cancer types from the perspective of tumour samples only, and subsequently leverages the expression of these genes observed in CCLs, to predict CCLs' cancer types or to compute a similarity‐to‐tumours score.

A distinctively different approach is TumorComparer (Sinha *et al*, 2021), which associates a weight to each multi‐omic feature while computing correlation scores between CCLs and primary tumours. An initial feature selection in this method is based on gene expression, CNAs and somatic mutations and outputs the 2,000 most variable features across all tumour types. Subsequently, TumorComparer assigns a weight to each feature in a 0–1 range based on their frequency of observation across primary tumours. Despite being a very useful framework due to the possibility of customising the feature weights (based on novel observational tumour data), assigning a bigger relevance to recurrent features in tumours might reward CCLs that are similar to very common cancer subtypes, possibly missing CCL that are good models of less recurrent ones. Still emphasising relevant features observed only in tumours, CELLector (Najgebauer *et al*, 2020) focuses on cancer functional events (CFEs) comprising recurrent mutated cancer genes, focal amplifications or deletions, and methylated gene promoters identified in patient tumours (Iorio *et al*, 2016). In cancer‐specific studies (Vincent *et al*, 2015; Jiang *et al*, 2016; Vincent & Postovit, 2017; Ronen *et al*, 2019; Batchu *et al*, 2020), features are instead filtered based on their variability observed jointly in the considered CCL and tumour data sets, weighting more features that are discriminative between *in vitro* models and patient tumours. Finally, in studies that include somatic mutations, the retained features are known (non‐synonymous) functional mutations present in both CCLs and tumours (Sinha *et al*, 2021; Najgebauer *et al*, 2020; Ronen *et al*, 2019; Zhao *et al*, 2017; Jiang *et al*, 2016) or a subset of the most relevant ones for a certain cancer type (Domcke *et al*, 2013).

In conclusion, as the aim of all the studies is to investigate the resemblance of CCLs to tumours, all the considered methods built on a wide range of features rather than reduced selections of oncogenic genes, but adopt disparate strategies to define a feature as informative.

### Multi‐omic integration and discordant cell line selection

Among the reviewed studies, 5 data types are considered for matching CCLs to primary tumours: gene expression (GE), somatic mutations (Mut), copy number alterations (CNAs), DNA methylation (HypMet) and protein expression (PE) (Table 2). In particular, we observe a tendency to include gene expression, either to investigate CCL‐tumour resemblance or as an additional means of validation, with 19 of 22 studies using gene expression from microarrays and bulk or single‐cell RNA‐seq. Only 12 studies consider more than one data type, among which 9 apply a multi‐omic integration method to combine multiple data modalities.

Specifically, CELLector (Najgebauer *et al*, 2020) combines multi‐omic cancer functional events (CFEs) encoded as binary matrices for CNA, Mut and HypMet. This allows accounting for most of the analysed patients when assembling the CELLector signatures (recurrent combination of CFEs) and hence led to large percentages of patient samples that are represented by at least one CCLs. That said, late integration is the most frequently adopted strategy, with CCLs/tumour mappings performed separately across individual omics and then combined at a later stage. For instance, studies based on an unsupervised method aggregate the rankings of CCL‐tumours similarities obtained from each omic type (Sun & Liu, 2015; Zhao *et al*, 2017; Sinha *et al*, 2021) then sum the omic‐specific correlations (Jiang *et al*, 2016), or just incorporate correlation scores together with selected mutation occurrences (Domcke *et al*, 2013). Supervised methods, such as HyperTracker (Salvadores *et al*, 2020) on the other hand, build a predictive model for each omics data type then combine the classification results. This strategy prevents capturing interactions between omics and a proper representation of underlying biological mechanisms. A more refined strategy, *intermediate integration*, is developed in Maui (Ronen *et al*, 2019) and MFmap (Zhang & Kschischo, 2021). Briefly, Maui considers GE, CNA, and Mut omics and builds a multimodal stacked variational autoencoder (VAE) to represent tumour samples and CCLs in a low dimension latent space, with the latent factors regarded as higher‐order genomic features. Similarly, MFmap transforms GE, CNA and Mut data into a low dimensional latent space via VAE that is then applied on the concatenated omics. This intermediate strategy enables a joint integration of all available data and projects them onto a common shared space, although less interpretable than the original molecular features. For example, the application of Maui to colorectal cancer (CRC) (Ronen *et al*, 2019) allowed the identification of a more refined subgrouping compared with the widely adopted CMS classification (Guinney *et al*, 2015), which associates with more pronounced differences in biological pathway activities and survival outcomes.

The use of multi‐omics also unveils discrepancies arising across omic‐specific CCL/tumour matching cases, emphasising different biological mechanisms controlling genetic, transcriptional or epigenetic changes. For instance, Jiang *et al* (2016) compute Pearson's correlation scores between CCLs and patient tumours in breast cancer across four different data modalities and find very different ranges, with GE exhibiting the highest values, followed by CNA, Mut and PE. In accordance with these results, TumorComparer (Sinha *et al*, 2021) finds GE as the data modality with the widest range of CCL/tumour similarity across 24 cancer types, followed by CNAs and Muts. In addition, Sinha *et al* (2021) showed that only 18 of 594 CCLs can be consistently assigned to a cancer type with a normalised rank > 0.9 (meaning that the CCL is more similar to the tumours type of origin than 90% of the considered panel) for all the 3 data omics, while several CCLs had a rank > 0.9 only for a single omic data modality. This highlights for example that CCLs with high gene expression similarity might retain tissue‐specific expression but lack characteristic genomic features (mutations or CNAs). Likewise confirmed by Zhao *et al* (2017), CCL similarity rankings resulting from different data omics are discordant. In CompHealth (Yu *et al*, 2019), transcriptional correlation scores for ovarian CCLs are compared with the results by Domcke *et al* (2013) that are instead based on CNAs together with informative Muts presence. This highlights a significant consistency (Spearman's corr. = 0.59, *P*‐value = 5.84e‐05). However, the two studies (Domcke *et al*, 2013; Yu *et al*, 2019) disagree on the CCL that are most transcriptionally and genomically similar to tumours, with CAOV4 ranking 1^st^ when considering transcriptomic data and only 9^th^ when considering genomics.

By implementing a supervised approach, HyperTracker (Salvadores *et al*, 2020) compares GE and HypMet classification results. One hundred and thirty‐one of 614 CCLs (silver set) are discordantly assigned to a cancer type but with the outcome resulting from analysing only one omic concordant to the annotated label. In the same study, 67 CCLs (undetermined set) are discordantly classified across all omics. Although partially discordant, the authors show that CCLs in the silver set could still be informative: joining CCLs in the silver and golden sets (composed of CCLs with correctly classified across all omics) and considering the result in the context of drug response datasets significantly changes drug response selectivity, in cancer types such as PAAD, and increased the number of significant pharmacogenomic associations. Finally, despite Celligner (Warren *et al*, 2021) is based on GE only, the authors compared their k‐NN assignments built on corrected GE with the k‐NN resulting from computing Jaccard similarity scores on CFEs (Muts, CNAs, HypMet) and found a similar classification accuracy (60% and 61% respectively). Nevertheless, CCL rankings change substantially, indicating that the different omics are representative of different processes and states.

In summary, the discrepancies arising from data underlying different states highlights the necessity of a proper multi‐omic integration to comprehensively capture tumour mechanisms.

## Extension to more complex *in vitro* and *in vivo* models

Complex models such as tumour organoids, patient‐derived xenografts (PDx) and genetically engineered mice (GEMM) have been compared with patient tumours in some of the studies we discuss in this Review. In particular, CancerCellNet (Peng *et al*, 2021) has also been applied to a collection of organoids, GEMM and PDx other than CCLs, to predict their cancer type and subtype in a supervised manner. Collectively, GEMM and organoids achieve the highest median correct classification scores in 4 of 5 tested cancer types, with organoids exhibiting the best classification rate, hence supposedly being the most appropriate tumour models. Indeed, compared with CCLs, GEMM are influenced by their native immune system and organoids benefit from cell–cell interactions arising from their 3D nature. Conversely, classification scores for PDxs demonstrated a bigger variability, with only few models yielding better scores than any of the organoids or GEMM. In the context of inter‐tumour heterogeneity, GEMM are the only models able to reflect a mixture of subtypes rather than modelling a single one, possibly due to a plasticity that is also influenced by the host environment. Although providing many insights, a proper comparison with different models derived from the same donor would be necessary to identify the most appropriate ones in representing patient tumours. Liu *et al* (2019a) also considered patient‐derived organoids for breast cancer and compared them to metastatic tumours, highlighting a better transcriptome resemblance compared with CCLs across breast cancer subtypes.

Beside highlighting the expected better performances of complex models in resembling tumours with respect to CCLs, these studies also demonstrate the adaptability of the CCLs versus tumours comparative analyses. While continuing to characterise increasingly larger collections of CCLs, the interest of the community is in parallel moving towards the development of large‐scale novel cancer models matched with original patient data. For instance, the recently established Human Cancer Models Initiative (HCMI) aims at creating a large collection of patient‐derived next‐generation cancer models that will include organoids and conditionally reprogrammed cells from diverse tumour subtypes and populations, with a particular attention to rare cancers that are at the moment widely unrepresented in *in vitro* models. Most importantly, collecting matched patients' samples and normal tissues will provide a unique opportunity to better understand the molecular changes introduced selectively in the *in vitro* model derivation phase.

Along the same lines, the PDXNet consortium has established more than 1,400 PDx samples with matched patient tumours, collecting sequencing data across multiple cancer types, and highlighting the suitability of this biobank for preclinical drug testing (Woo *et al*, 2021).

Looking at rare tumour models, a recent paediatric high‐grade glioma PDx collection comprising 21 models that included DNA methylation, mutation, and gene expression profiles was assembled from matched patient tumours (He *et al*, 2021). Analogous efforts are made to properly represent the interactive effect between *in vitro* models and TME, although so far at a smaller scale. For example, co‐culturing strategies for 3D patient‐derived organoids have been developed to include stromal and immune components (Neal *et al*, 2018; Tsai *et al*, 2018) and cell culture media have been modified to resemble human plasma (Cantor *et al*, 2017; Rossiter *et al*, 2021) or to include cancer‐associated fibroblasts (Cheteh *et al*, 2017).

## Future directions

### Advanced multi‐omic integration

Up to now, computational methods have generally focused on using particular feature types or employing simple strategies to combine features across modalities such as late or early integration.

However, different omics modalities encode for distinct but complementary information in cancer biology, with the genome regarded as the first affected layer when the tumour originates that constantly undergoes selective pressure, and the epigenome and transcriptome as more malleable and consequential states that are disrupted both by oncogene mutations and the environment. Because of the intricate interplay among the different biological components, and considering the large available collection of wide molecular characterizations, now more than ever we need integration methods able to learn a unified representation of cancer features. With a proper multi‐omic integration, the mapping of CCLs to tumours would focus on resembling as much as possible the underlying tumour‐specific mechanisms rather than the approximation of a single state.

Such considerations necessitate approaches that address the omic‐specific technical challenges (e.g. batch effects), while flexibly and efficiently integrating information across data modalities and potentially capturing variable information content across diverse data sets/cancer types. In this direction, Maui (Ronen *et al*, 2019) and MFmap (Zhang & Kschischo, 2021) are the only methods among the reviewed studies that adopt a joint representation of the multiple data modalities via a VAE approach. In general, similar challenges have emerged in the context of single‐cell methodologies and advanced methods have been proposed, focussing on the integration of multimodal simultaneously measured data (e.g. CITE‐seq or SHARE‐seq) such as MOFA+, totalVI and WNN (Argelaguet *et al*, 2020; Gayoso *et al*, 2021; Hao *et al*, 2021). For example, MOFA+ and totalVI, built on variational inference, are highly efficient in a large‐scale context and can incorporate multiple data omics measured across different batches while accounting for noise and technical biases of each omic modality. In the context of bridging CCLs and tumours, these methods could be adapted regarding different batches as tumour and *in vitro* model division.

Finally, none of the studies reviewed here employ a hierarchical integration strategy combining the different omic components from a regulatory point of view, despite the clear evidence of genetic drift leading to changes in gene expression and consequently drug screenings (Ben‐David *et al*, 2018). This sort of approach could reveal complex mechanisms that would otherwise remain undetected, building a regulatory network of a tumour and then mapping single CCLs in it leveraging conserved and shared mechanisms. Indeed, from a holistic point of view, Webber *et al* (2018) highlighted that inferred gene regulatory networks for tumours compared with those built on CCLs include preserved modules that are highly predictive of therapeutic response.

### Transfer learning

With the goal of developing personalised treatments, translating *in vitro* measured key phenotypes to patient tumours rely on our ability to understand these relationships. In this context, transfer learning is a powerful tool to leverage drug sensitivity data in CCLs training machine learning models and transfer those predictions to patient tumours. Particularly prominent in the framework of image classification at pathologist level accuracy (Esteva *et al*, 2017; Coudray *et al*, 2018) and molecular subtyping from gene expression (Sevakula *et al*, 2019), transfer learning relies on the assumption that a predictive feature learned in a certain domain can be applied and adapted to a different but analogous one, even of limited sample size. Although promising, the main challenges are rooted in the inconsistencies between protocols and techniques used in different studies possibly leading to batch effects hard to be generalised, for example the way drug response is assessed. Specifically, transfer learning methods in model‐tumour context for drug sensitivity should consider fundamental differences between their genomic profiles, ultimately aiming at understanding which results can be robustly transferred, and how to optimally adjust model predictions.

In this context, pioneer works aim at learning a shared structure that can be leveraged for drug response prediction on patient tumour. For example, Geelher et al (Geeleher *et al*, 2014) used batch correction (ComBat) on gene and miRNA expression data between *in vitro* model and patient tumour to build a predictive model of drug sensitivity on CCLs and further validated the predicted drug response in primary tumours with respect to known clinical trial results. More recent methodologies such as PRECISE (Mourragui *et al*, 2019) and TRANSACT (Mourragui *et al*, 2021) first learn a shared feature subspace (linear and non‐linear, respectively) and then use it to build a predictive model for drug response. Sharifi‐Noghabi *et al* (2020) proposed an adversarial inductive transfer learning method that focuses on discrepancies in both gene expression (input) and drug response (output), adapting both aspects in the two different domains.

Across model domains, Ma *et al* (2021) developed a transfer learning method (few‐shot learning) based on a neural network trained to identify relevant input features (mutations and gene expression) for cell‐based screenings by optimising their transferability to patient‐derived tumour cells and patient‐derived xenografts, in a small sample size setting for the target domain. They also evaluated the ability of their transfer learning model to predict CRISPR‐Cas9 screening outcomes of CCLs considering as target domain another tissue with reduced sample size. A similar approach, from one large tissue to an underrepresented one, showed promising results in terms of drug synergy prediction (Kim *et al*, 2021). Finally, Villemin *et al* (2021) used a transfer learning strategy to identify biomarkers for basal A and B subtypes in breast CCLs in a supervised way, while iteratively adapting this model prediction to the tumour and integrating those that are classified with confidence.

### Leveraging single‐cell data sets

The reviewed studies do not investigate intra‐tumour heterogeneity (with few exceptions (Vincent & Postovit, 2017; Peng *et al*, 2021)) neither do they address population heterogeneity in *in vitro* models. Nevertheless, large‐scale single‐cell genomics investigation could potentially help with resolving relationships between *in vitro* models and tumour cell populations more precisely and with greater resolution. First, comparing *in vitro* models and tumours at the single‐cell level could mitigate the limitations arising from tumour purity by comparing CCLs solely with malignant cells. Second, single‐cell studies could enable a more fine‐grained mapping of the relationship of the subclonal populations and cell states in heterogeneous tumour populations to representative *in vitro* models, effectively delineating each tumour as a mixture of *in vitro* models. For example, Gambardella *et al* (2022) created a single‐cell atlas of 32 breast CCLs and showed that the single‐cell transcriptional profile from a single patient could be mapped into the *in vitro* model atlas to assign a CCL model to each patient's cells. Strikingly, the tumours were found to be highly heterogeneous as none was mapped into a single CCL and they were overall represented by a mixture of models.

Pan‐cancer tumour single‐cell atlas studies could also reveal the predominant sources of intra‐tumour heterogeneity and their relationship to different *in vitro* models. Indeed, pan‐cancer single‐cell characterisation of CCLs (Kinker *et al*, 2020) has revealed that many of the recurrent drivers of transcriptional heterogeneity in tumours (preprint: Gavish *et al*, 2021) are also observed in CCLs, suggesting that individual CCLs might be evaluated as models for different components of intra‐tumour heterogeneity.

More generally, the development of large single‐cell atlases for both *in vitro* models and tumour patients offer a unique opportunity to create an integrated reference, enabling direct comparisons of tumours and models with single‐cell resolution.

### Increasing interpretability

Despite the advantages in obtaining a similarity score representative for each cell type, the majority of the methods developed so far do not investigate the underlying factors that are driving these similarities (with the exception of CELLector (Najgebauer *et al*, 2020)). To adequately interpret the results arising from a matching procedure, we need interpretable methods, with the goal of understanding the shared mechanisms between tumours and a CCL and possibly their connection to treatment responses or genetic dependencies. Network and pathway approaches could be leveraged to enhance interpretability. These approaches use information from Reactome, Gene Ontology and protein–protein interaction databases to guide a more biologically relevant comparison of the underlying cell states.

### Filling the gap in ancestry representation

As previously noted, currently available *in vitro* models are not representative of the human population and are mostly skewed towards European and Asian cohorts (Dutil *et al*, 2019). New *in vitro* models should be generated keeping in mind panel heterogeneity (e.g. HCMI), as ethnicity is reflected in differences in mutation frequency and transcriptional signatures that may cover specific mechanisms that are not present in the current panel. On the contrary, before a reasonable size can be reached to reliably build models for each ancestry and because of the heterogeneity of the concept itself, it would be important to also address this aspect in new computational approaches, for example via linear mixed models, a concept established in genome‐wide association studies (Loh *et al*, 2015).

## Concluding remarks

The methods developed so far guided the evaluation of CCLs as proper tumour models, identified problematic cell lines showing putative misclassification as well as undifferentiation, directed towards CCLs representative of tumour subpopulation (known or estimated) and pointed out gaps in *in vitro* models. These studies provide a systematic framework to assess tumour patient populations lacking *in vitro* models and allow guiding gap‐filling efforts, consequently generating new hypotheses for under‐represented groups.

In all the reviewed studies, the assessment of the *in vitro* CCL as a proper model is built upon its closeness to the selected tumour population based on genomic features. Although resembling the tumour as close as possible can reveal both shared mechanisms and aid the translational medicine process, a feature‐specific approach could be preferred for targeted purposes. For example, *in vitro* models of microsatellite instability might be useful to identify dependencies, regardless of their similarity to primary tumours. In addition, the field still lacks methods capable of properly handling and integrating multi‐omic datasets. Matching CCLs to primary tumour from a more “global” perspective can only be addressed via methods that take into consideration the landscape of available multi‐omic data and the discrepancies observed across individual omic comparisons.

Looking forward, characterisation at the single‐cell level could circumvent some of the intrinsic limitations of CCLs repurposing them as more close models of specific tumour cellular states and intra‐tumour heterogeneity components. Despite all the limitations, we envision that the large data collection available for CCLs, readily available to the entire community via public repositories, will still be widely used in the future to model and understand cancer mechanisms and to aid early anti‐cancer drug discovery. On the contrary, large panels of newly developed models will help recapitulating and validating mechanisms that are impossible to observe in the actual available 2D models, among which genomic features characteristic solely of the model establishment, three‐dimensional assembly, the interplay with TME, drug assimilation and half‐life in blood streams.

Developing computational methods to align and compare existing or newly generated cancer models and tumour patients will continue to play a pivotal role for the effective use of these models in investigating the biology of cancer, as well as for contributing to the realisation of the personalised medicine paradigm.

## Author contributions

**Lucia Trastulla:** Conceptualization; visualization; writing – original draft; writing – review and editing. **Javad Noorbakhsh:** Writing – original draft. **Francisca Vazquez:** Writing – original draft; writing – review and editing. **James McFarland:** Writing – original draft; writing – review and editing. **Francesco Iorio:** Conceptualization; supervision; funding acquisition; visualization; writing – original draft; writing – review and editing.

## Disclosure and competing interests statement

F.I. receives funding from Open Targets, a public‐private initiative involving academia and industry, and performs consultancy for the joint AstraZeneca‐CRUK functional genomics centre and for Mosaic Therapeutics. J.M. receives funding from Dependency Map Consortium. F.V. receives funding from Dependency Map Consortium and Novo ventures. J.N. is a shareholder of Kojin Therapeutics.
